# STRprofiler: efficient comparisons of short tandem repeat profiles for biomedical model authentication

**DOI:** 10.1093/bioinformatics/btae713

**Published:** 2024-11-26

**Authors:** Jared M Andrews, Michael W Lloyd, Steven B Neuhauser, Margaret Bundy, Emily L Jocoy, Susan D Airhart, Carol J Bult, Yvonne A Evrard, Jeffrey H Chuang, Suzanne Baker

**Affiliations:** Department of Developmental Neurobiology, St. Jude Children's Research Hospital, Memphis, TN 38105, United States; The Jackson Laboratory, Bar Harbor, ME 04609, United States; The Jackson Laboratory, Bar Harbor, ME 04609, United States; The Jackson Laboratory, Sacramento, CA 95838, United States; The Jackson Laboratory, Sacramento, CA 95838, United States; The Jackson Laboratory, Bar Harbor, ME 04609, United States; The Jackson Laboratory, Bar Harbor, ME 04609, United States; Leidos Biomedical Research, Inc, Frederick National Laboratory for Cancer Research, Frederick, MD 21701, United States; The Jackson Laboratory for Genomic Medicine, Farmington, CT 06032, United States; Department of Developmental Neurobiology, St. Jude Children's Research Hospital, Memphis, TN 38105, United States

## Abstract

**Summary:**

Short tandem repeat (STR) profiling is commonly performed for authentication of biomedical models of human origin, yet no tools exist to easily compare sets of STR profiles to each other or an existing database in a high-throughput manner. Here, we present STRprofiler, a Python package, command line tool, and Shiny application providing methods for STR profile comparison and cross-contamination detection. STRprofiler can be run with custom databases or used to query against the Cellosaurus cell line database.

**Availability and implementation:**

STRprofiler is freely available as a Python package with a rich CLI from PyPI https://pypi.org/project/strprofiler/ with source code available under the MIT license on GitHub https://github.com/j-andrews7/strprofiler and at https://zenodo.org/records/10989034. A web server hosting an example STRprofiler Shiny application backed by a database with data from the National Cancer Institute-funded PDXNet consortium and The Jackson Laboratory PDX program is available at https://sj-bakerlab.shinyapps.io/strprofiler/. Full documentation is available at https://strprofiler.readthedocs.io/en/latest/.

## 1 Introduction

Human cell lines and patient-derived xenograft (PDX) models are invaluable and commonly used tools for biological and pharmaceutical research (Rosfjord *et al.* 2014, Mirabelli *et al.* 2019, Abdolahi *et al.* 2022), but they are at risk for cross-contamination and mislabeling. Misidentified cell lines have been a known issue for over 50 years (Gartler 1968), leading to erroneous conclusions and a massive waste of resources. A 2021 study estimating the financial impact of the usage of Intestine 407 and HEp-2 found that over $900 million was spent to publish nearly 10 000 articles in which these two HeLA-contaminated cell lines were used (Korch and Capes-Davis 2021). A 2017 study found 32 755 published articles using known misidentified cell lines, which were cited by roughly 500 000 other papers (Horbach and Halffman 2017). Worryingly, the authors also found that the rate of contaminated cell line usage is not decreasing over time and is geographically widespread.

Numerous studies have identified 15%–45% misidentification across hundreds of cell lines (Ye *et al.* 2015, Drexler *et al.* 2017, Huang *et al.* 2017, Gu *et al.* 2022). Despite this, many studies are published each year using known contaminated or misidentified cell lines (Buehring *et al.* 2004, Makowska *et al.* 2024). Due to these alarming trends, increasing numbers of journals are implementing cell line authentication policies, including *Nature*, *Cell Press*, *EMBO Press*, the *International Journal of Cancer*, and the *American Association for Cancer Research*. A 2022 study of the mandatory cell line authentication at the *International Journal of Cancer* revealed that at least 5% of human cell lines in submitted manuscripts between July 2018 and June 2021 were misidentified (Souren *et al.* 2022).

As with cell lines, PDX models have been used extensively in pharmacology and preclinical drug testing. Ensuring that patient-derived model samples are not mishandled and avoiding misidentified results is of equal importance for such models; however, misidentification in PDX models across studies has not been as widely documented. Historically, the validation of PDX models has primarily been concerned with reproducing primary disease state (Rosfjord *et al.* 2014), but determination of the genetic identity of models is strongly recommended (Mattar *et al.* 2018).

Ensuring the authenticity and purity of cell lines and patient-derived models is critical for the integrity of scientific research and efficient use of resources. Short tandem repeat (STR) profiling is the recommended method for model authentication (Masters *et al.* 2001, Capes-Davis *et al.* 2013, El-Hoss *et al.* 2016, Mattar *et al.* 2018), which requires the comparison of STR profiles between a test sample and previously generated reference sample. Generation of cell lines and PDX models from primary samples is expensive and time-consuming, so it is best practice for groups that generate and utilize them to serially profile them over time or after transfer between groups to ensure their authenticity. Doing so also provides a way to measure genetic drift over time in culture, which can be significant for certain lines (Parson *et al.* 2005). Additionally, the NCI Patient-Derived Models Repository (PDMR) requires STR profiling before submission of material for banking and recommends repeated profiling over the course of any PDX-based experimental process. It is also important to note that natural intra-model allelic variation in STR profiles does exist (Zhang *et al.* 2018). Such variation is most commonly seen as allelic drop-out due to loss of heterozygosity as demonstrated in models with microsatellite instability (Poetsch *et al.* 2004, Vauhkonen *et al.* 2004). Documentation of such intra-model variation may be important for model validation if there is a concern of cross-contamination.

Despite the growing necessity of this practice, no software exists to easily and quickly compare arbitrary STR profiles to identify matches or potential contaminants. STRprofiler was developed to fill this critical gap in the efforts to use model systems to guarantee reproducible results.

## 2 Methods and results

STRprofiler is a Python package and Shiny application for comparing short tandem repeat (STR) profiles. Profiles are frequently generated after model generation and throughout maintenance to authenticate their identity, determine sample mixing, and measure genetic drift during culture and PDX passaging. STRprofiler provides a simple command line interface (CLI) or interactive web application for comparing STR profiles and detecting sample mixing, which can prove a time-consuming and tedious task when performed manually. While there exist comprehensive STR profile databases and tools, such as the Cellosaurus STR Similarity Search Tool (CLASTR; https://www.cellosaurus.org/str-search/) (Robin *et al.* 2020), that allow comparisons to established and published cell lines in a low-throughput manner, these databases and tools are inadequate for research groups that generate, utilize, and maintain their own models not yet available through public repositories. STRprofiler allows researchers to compare all their STR profiles quickly and easily to each other or to existing databases, running on collections of thousands of STR profiles in seconds on a typical laptop.

STRprofiler was designed to be simple to use and interpret. It utilizes flexible and straightforward STR profile formats as input, supporting popular STR platforms such as PowerPlex. For all input STR profiles, each profile will be compared to every other profile or to each sample in the database provided and scored for similarity using the Tanabe (Tanabe *et al.* 1999), Masters (versus query), and Masters (versus reference) algorithms (Masters *et al.* 2001):

- Tanabe, also known as the Sørenson–Dice coefficient:
  $$\mathrm{Score}\;=\;\frac{2\;\times \;\mathrm{no}.\;\mathrm{shared}\;\mathrm{alleles}}{\mathrm{no}.\;\mathrm{query}\;\mathrm{alleles}\;+\;\mathrm{no}.\;\mathrm{reference}\;\mathrm{alleles}\;}$$
- Masters (versus query):
  $$\mathrm{Score}\;=\;\frac{\mathrm{no}.\;\mathrm{shared}\;\mathrm{alleles}}{\mathrm{no}.\;\mathrm{query}\;\mathrm{alleles}}$$
- Masters (versus reference):
  $$\mathrm{Score}\;=\;\frac{\mathrm{no}.\;\mathrm{shared}\;\mathrm{alleles}}{\mathrm{no}.\;\mathrm{reference}\;\mathrm{alleles}}$$

The Masters and Tanabe formulae used with an 80% match threshold (the default in STRprofiler) have been demonstrated to accurately identify matching profiles in 98%–99% of cases, with most failures arising in models with known variation due to microsatellite instability (Capes-Davis *et al.* 2013). In such models, use of additional loci or validation with alternative approaches (e.g. SNP analysis) may be necessary. To identify potential sample contamination, STRprofiler flags profiles with three or more alleles detected for three or more loci for further investigation. The Masters algorithms are particularly useful for determining the potential contaminating samples when unintentional mixing occurs.

STRprofiler returns two types of output files. The first is a summary file containing a record for each STR profile, its top matches with the Tanabe algorithm, all matches for each algorithm that meet the scoring threshold, and a flag for potential sample mixing based on the number of markers with three or more alleles detected. The second is a profile-specific file with that profile queried against all others. This file allows for closer interrogation of samples with potential mixing or genetic drift.

STRprofiler also contains a command to compare profiles to the Cellosaurus database using the CLASTR API, enabling convenient, high-throughput access to the most expansive STR profile database available (Robin *et al.* 2020). This functionality is available from both the command line and through the Shiny application, returning rich results in Excel format with links out to the relevant cell line pages on Cellosaurus.

In conclusion, STRprofiler fills a critical gap in ensuring the integrity and authenticity of models broadly used in biomedical research. It aids in the prevention of erroneous data generation and interpretation, which is a major concern in this field.

## Data Availability

No new data were generated or analysed in support of this research. However, the data underlying the example STRprofiler Shiny application are publicly available from The Jackson Laboratory PDX Program (https://tumor.informatics.jax.org/mtbwi/pdxSearch.do) and the NCI's Patient-Derived Model Repository (https://pdmr.cancer.gov/).
